# Synthesis and photophysical characteristics of polyfluorene polyrotaxanes

**DOI:** 10.3762/bjoc.11.288

**Published:** 2015-12-21

**Authors:** Aurica Farcas, Giulia Tregnago, Ana-Maria Resmerita, Pierre-Henri Aubert, Franco Cacialli

**Affiliations:** 1 Supramolecular Chemistry Group, ‘‘Petru Poni’’ Institute of Macromolecular Chemistry, Grigore Ghica Voda Alley, 700487-Iasi, Romania; 2 London Centre for Nanotechnology and Department of Physics and Astronomy University College London, Gower Street, London WC1E 6BT, UK; 3 Laboratoire de Physicochimie des Polymères et des Interfaces (EA 2528), Institut des Matériaux, Université de Cergy-Pontoise, F-95031 Cergy-Pontoise Cedex, France

**Keywords:** energy band gaps, fluorescence lifetimes, permethylated cyclodextrins, polyfluorene, polyrotaxanes

## Abstract

Two alternating polyfluorene polyrotaxanes (**3·TM-βCD** and **3·TM-γCD**) have been synthesized by the coupling of 2,7-dibromofluorene encapsulated into 2,3,6-tri-*O*-methyl-β- or γ-cyclodextrin (TM-βCD, TM-γCD) cavities with 9,9-dioctylfluorene-2,7-diboronic acid bis(1,3-propanediol) ester. Their optical, electrochemical and morphological properties have been evaluated and compared to those of the non-rotaxane counterpart **3**. The influence of TM-βCD or TM-γCD encapsulation on the thermal stability, solubility in common organic solvents, film forming ability was also investigated. Polyrotaxane **3·TM-βCD** exhibits a hypsochromic shift, while **3·TM-γCD** displays a bathochromic with respect to the non-rotaxane **3** counterpart. For the diluted CHCl_3_ solutions the fluorescence lifetimes of all compounds follow a mono-exponential decay with a time constant of ≈0.6 ns. At higher concentration the fluorescence decay remains mono-exponential for **3·TM-βCD** and polymers **3**, with a lifetime τ = 0.7 ns and 0.8 ns, whereas the **3·TM-γCD** polyrotaxane shows a bi-exponential decay consisting of a main component (with a weight of 98% of the total luminescence) with a relatively short decay constant of τ_1_ = 0.7 ns and a minor component with a longer lifetime of τ_2_ = 5.4 ns (2%). The electrochemical band gap (Δ*E*_g_*)* of **3·TM-βCD** polyrotaxane is smaller than that of **3·TM-γCD** and **3**, respectively. The lower Δ*E*_g_ value for **3·TM-βCD** suggests that the encapsulation has a greater effect on the reduction process, which affects the LUMO energy level value. Based on AFM analysis, **3·TM-βCD** and **3·TM-γCD** polyrotaxane compounds exhibit a granular morphology with lower dispersity and smaller roughness exponent of the film surfaces in comparison with those of the neat copolymer **3**.

## Introduction

Over the last decades, conjugated polymers (CPs) have been actively investigated as an alternative to conventional inorganic materials in many electronic applications due to their low cost and easy processability [1–6]. Among the various CPs, polyfluorenes (PFs) have been intensively studied as emitting materials owing to their pure blue emission [7–11]. However, some major drawbacks for their use are their high ionization potential associated with low photoluminescence (PL) efficiency, their rather large band gap and facile photochemical degradation [12–13]. Different strategies have been employed in view to reduce these undesirable effects, e.g., the synthesis of copolymers [14–17], block copolymers [18], the introduction of donor (D) and acceptor (A) moieties [19–21], or bulky substituents at the C-9 position of the fluorene units [22–24], incorporating PF moieties into zeolites [25], nanochannels [26], or by wrapping with amylose [27]. The past decade has witnessed remarkable innovations and progress in polymer science, including the field of supramolecular science as a complementary field, which offers great opportunity for new concepts, new materials with unique properties, and novel practical applications. The construction of polyrotaxane architectures has an impact on the polymer-chain behavior and subsequently generates smart functional polymeric materials [28–31]. Polyrotaxanes with conjugated polymers have attracted considerable attention over the last decades due to their architectures and topologies, but mostly because they provide an efficient strategy to achieve an “insulation” of individual molecular wires [30]. Additionally, the synthesis of such structures makes it possible to tune a large number of physicochemical properties of conjugated polymers [16–20,26–38]. The first step in the preparation of conjugated polyrotaxanes is the threading of macrocyclic compounds (hosts) onto linear chains (guests), when a thermodynamically unstable inclusion complex (IC) is obtained. A wide variety of host molecules have the ability to encapsulate the π-conjugated backbones into their cavities based on intermolecular interactions, and thus leading to ICs. Cyclodextrins (CDs) are by far the most intensively investigated macrocyclic molecules in the synthesis of such supramolecular architectures [39]. The second most investigated group of host molecules in the synthesis of conjugated polyrotaxanes is comprised of chemically-modified CDs. They are less hydrophilic than native CDs, and should exhibit a significantly increased ability to bind aromatic guests through ionic, ion-dipole, as well as hydrophobic interactions. CD liphophilic derivatives are more soluble in non-polar solvents and water and exhibit lower propensity to aggregate than native CDs [40–42]. Considering that larger hydrophobic CD surfaces can lead to increased interactions with the hydrophobic aromatic guest, several types of permodified CD derivatives, such as 2,3,6-tri-*O*-methyl-βCD (TM-βCD) or 2,3,6-tri-*O*-methyl-γCD (TM-γCD) have been synthesized in the course of our investigations.

**Scheme 1:**
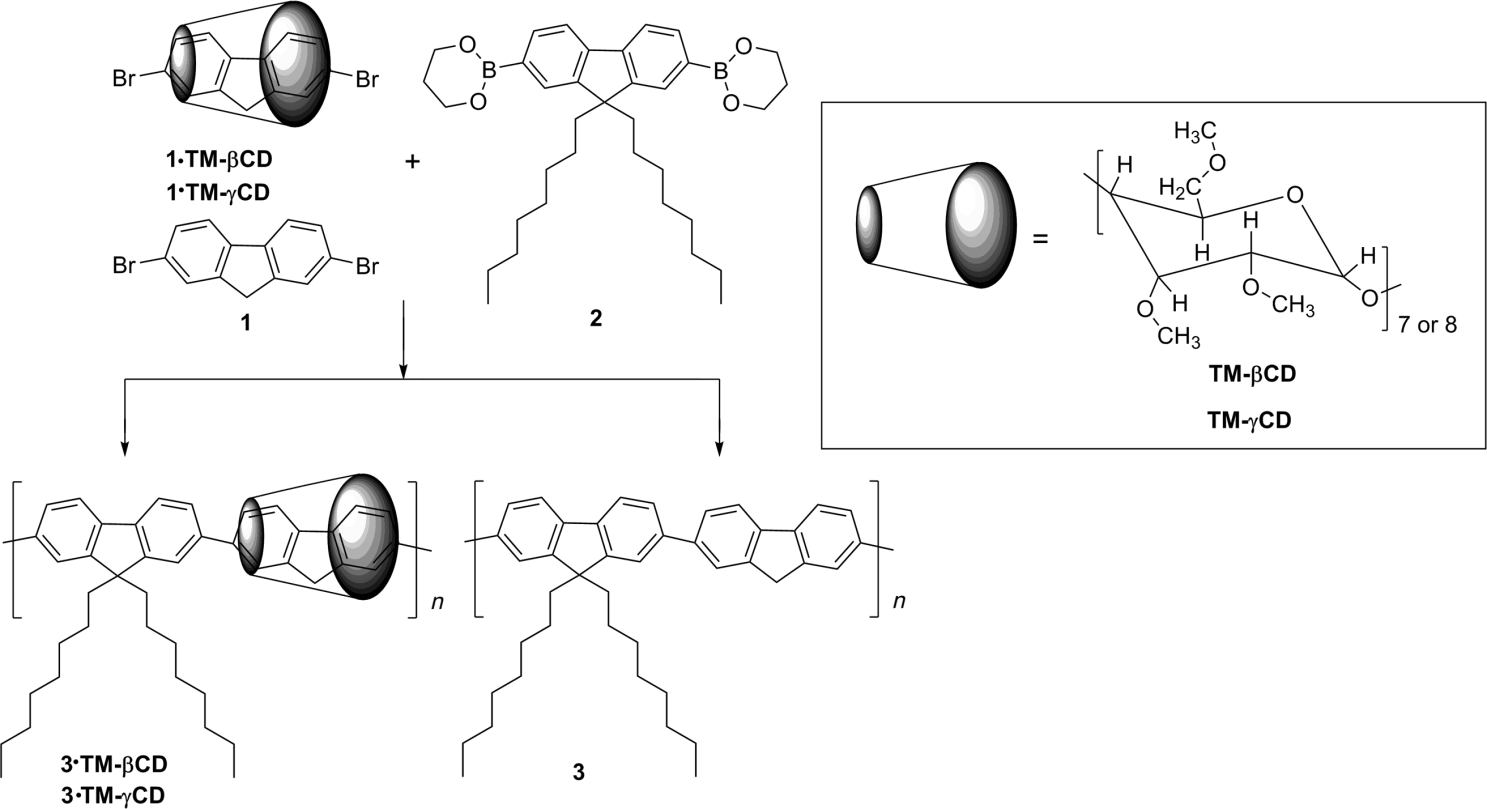
Synthetic route of **3·TM-βCD** and **3·TM-γCD** polyrotaxanes, and the non-rotaxane counterpart **3**.

With a view to better understand the influence of TM-βCD and TM-γCD encapsulations on the photophysical properties of PF, poly[2,7-(9,9-dioctylfluorene)-*alt*-2,7-fluorene/TM-βCD)] (**3·TM-βCD**) and poly[2,7-(9,9-dioctylfluorene)-*alt*-2,7-fluorene/TM-γCD)] (**3·TM-γCD**) polyrotaxanes have been synthesized. Thus, **3·TM-βCD** and **3·TM-γCD** have been obtained through the Suzuki cross-coupling reaction of 2,7-dibromofluorene (**1**) encapsulated into TM-βCD or TM-γCD cavities (**1·TM-βCD** and **1·TM-γCD)** with 9,9-dioctylfluorene-2,7-diboronic acid bis(1,3-propanediol) ester (**2**), as bulky stopper units [43]. The thermal, surface morphology, optical as well electrochemical characteristics of both polyrotaxanes were compared to those of the non-threaded **3** counterpart, Scheme 1.

## Results and Discussion

In continuation of our interest on the exploration of photophysical properties of PF copolymers by supramolecular encapsulation, we have performed the present study by using liphophylic CD derivatives, such as TM-βCD and TM-γCD instead native β- or γCD [43–45], or TMS-γCD [46]. Therefore, **3·TM-βCD** and **3·TM-γCD** polyrotaxanes were synthesized by Suzuki coupling of **1** being in the form of its IC (**1·TM-βCD** or **1·TM-γCD)** with **2** followed by the termination of the growing chains by bromobenzene, Scheme 1. To have the reference the neat copolymer **3** was also synthesized by coupling **1** with **2** under similar reaction conditions (Scheme 1).

TM-βCD and TM-γCD macrocyclic molecules were prepared according to previously reported procedures [47]. **1·TM-βCD** and **1·TM-γCD** were synthesized in water by using a 2:1 molar ratio of macrocycles and monomer **1**. The synthesis of **1**·**TM-βCD** or **1**·**TM-γCD** in polar protic solvents is driven by hydrophobic interactions in combination with electrostatic, van der Waals or π–π interactions. In comparison, in polar aprotic solvents such as DMF, THF relies mostly on host–guest specific interactions, such as dispersion or dipole–dipole interactions.

As results of the encapsulation into TM-βCD and TM-γCD cavities compared to native CDs [43–45], i.e., the use of toluene as solvent medium instead of a 3:1 v/v toluene/DMF mixture led to compounds soluble in toluene, THF, CH_2_Cl_2_ (DCM), and CHCl_3_. **3·TM-βCD** due to its higher coverage showed 7% water solubility. In addition, better optical quality films could be prepared by spin-coating from **3·TM-βCD** and **3·TM-γCD** THF, DCM, and CHCl_3_ solutions.

The investigated guest **1** proved binding ability to the hosts TM-βCD and TM-γCD, according to our determination of constant stability (*K*_s_), which was performed by UV–vis absorption in CHCl_3_. Changes in the absorption intensity of **1** at 321 nm in the presence of increasing concentrations of TM-βCD or TM-γCD provides the values of *K*_s_, Figures S1 and S2 in Supporting Information File 1. The analysis data shows that *K*_s_ could be approximately around 580 ± 100 and 160 ± 30 M^−1^ for **1·TM-βCD** and **1·TM-γCD**, respectively. *K*_s_ values of TM-βCD encapsulation were higher than that of TM-γCD, due to its more favorable dimensional compatibility.

Characterization of these compounds has been performed using FTIR and NMR spectroscopy. Figure S3 in Supporting Information File 1 gives the FTIR spectra of both polyrotaxanes and the reference **3**. FTIR of encapsulated compounds **3·TM-βCD** and **3·TM-γCD** reveals a distinct vibration peaks located in 1159–1042 cm^−1^ region due to the presence of TM-βCD or TM-γCD, whereas the reference **3** does not show any absorption peaks in this interval. Consequently, the disappearance of the characteristic peaks in 1159–1042 cm^−1^ region in the FTIR spectrum of reference **3** evidences the presence of macrocycles on **3·TM-βCD** and **3·TM-γCD**, well consistent with ^1^H NMR results.

**Figure 1:**
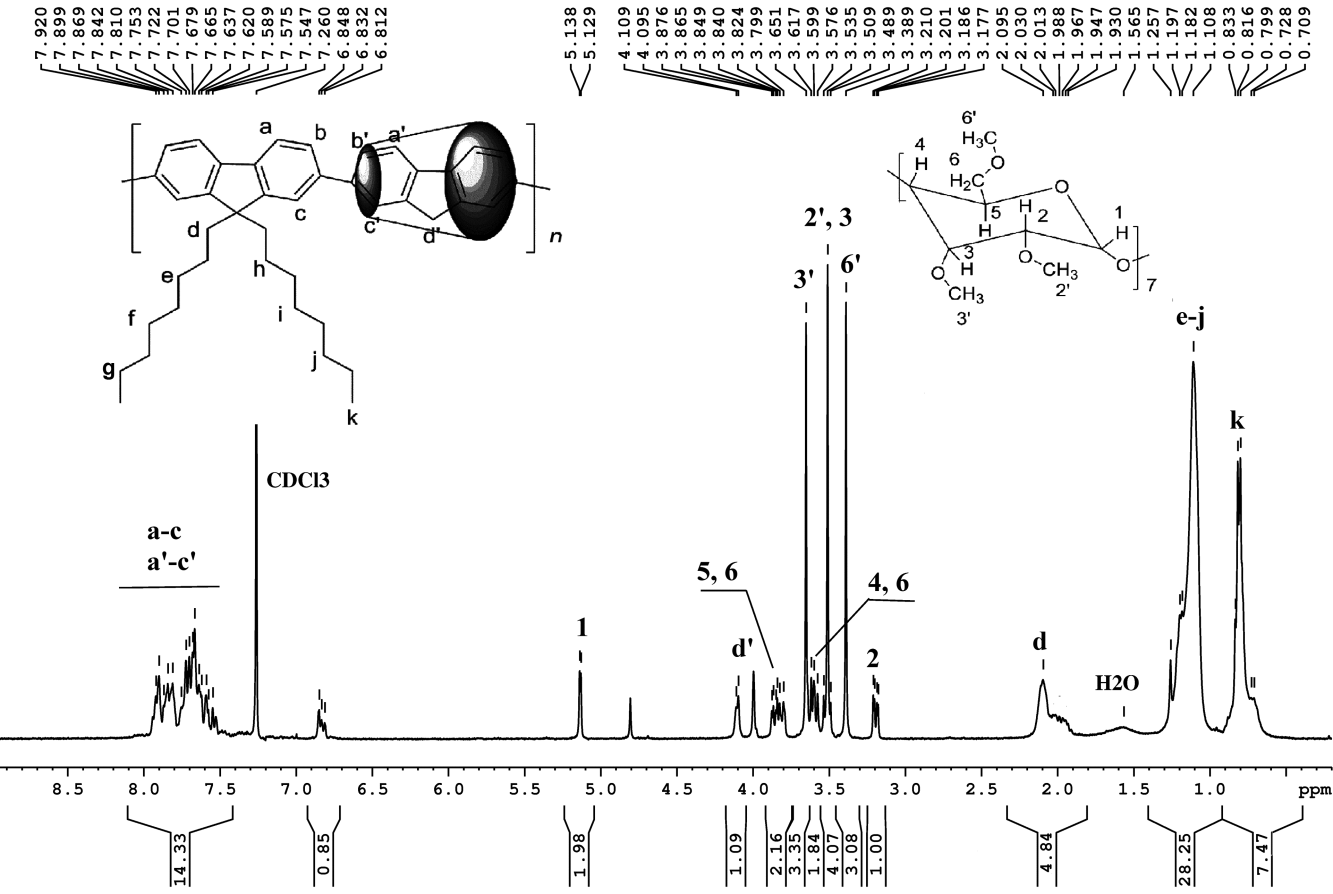
^1^H NMR spectrum of the polyrotaxane **3·TM-βCD** copolymer in CDCl_3_.

As expected, the ^1^H NMR spectrum of **3·TM-βCD** polyrotaxane exhibits correlation peaks of both H3 and H5 protons of TM-βCD with those methylene protons (H_d_) protons of monomer **2**, and all the characteristic protons have been identified, Figure 1. Figures S4–S7 in Supporting Information File 1 show the ^1^H NMR and ^13^C NMR spectra of **3·TM-γCD** and the reference **3**.

The resonance peak of the d proton from monomer **2** is upfield shifted by more than 0.06 ppm in the polyrotaxane **3·TM-βCD** compared to those of the non-rotaxane **3** counterpart, as shown in Figure 1 and Figure S4 in Supporting Information File 1. The resonance peaks of a–c and a’–c’ protons of **3·TM-βCD** rotaxane copolymer are also upfield shifted by 0.05 ppm as compared to those of the non-rotaxane homologue, while all protons of the TM-βCD macrocycle are shifted by more than 0.07 ppm. Comparing the integrals of d' protons from monomer **1** to those corresponding to H1 protons of TM-βCD, the average number of coverage per repeating unit has been calculated. By using the ratio of the integrated area of the H1 from TM-βCD (5.13–5.12 ppm, I_H-1_) and the methylene proton peaks of the monomer **1** (4.11–4.09 ppm, I_d’_); (I_H-1_/7)/(I_d’_/2) the coverage ratio was found to be of about 0.26 (i.e., ca. 26% coverage) suggesting that about every three structural unit was threaded with TM-βCD macrocycle. However, compared with native CD [43,45], ^1^H NMR results suggest poor hydrophobic–hydrophobic interactions of molecule **1** towards TM-βCD. Unfortunately, as a consequence of the low *K*_s_ of **1·TM-γCD**, the polyrotaxane **3·TM-γCD** presented only 11% coverage. The physical properties of the investigated copolymers are listed in Table 1.

**Table 1:** Physicochemical characteristics of **3**, **3·TM-βCD** and **3·TM-γCD**.

Sample	*M*_n_^a^	*M*_w_/*M*_n_^b^	Coverage^c^ (%)	*T*_g_^d^ (°C)

**3**	27900	1.83	—	88
**3·TM-βCD**	24300	1.94	26	104
**3·TM-γCD**	20100	2.24	11	96

^a^Number average molecular weight determined by GPC, THF, Polystyrene (Pst) standards. ^b^Polydispersity index. ^c^Average number of macrocycles /structural units, determined from ^1^H NMR analysis. ^d^Glass-transition temperature estimated from the second-heating DSC measurements.

The polydispersity index (*M*_w_/*M*_n_) and molecular weight distributions (*M*_n_) of polymers obtained by gel permeation chromatography (GPC) analysis using Pst standards and THF as eluent, are presented in Table 1. Two things should be noted here concerning the lower *M*_n_ of **3·TM-βCD** and **3·TM-γCD** polyrotaxanes than that of the neat copolymer **3**. Firstly, the less ability of ester groups from molecule **2** to partially penetrate the macrocyclic cavities in the condensation reaction due to the sterical hindrance of methyl groups [48]. Secondly, could be assigned to the differences of the hydrodynamic radii of the polyrotaxane rod*-*like backbones and standards. Furthermore, the polarity and backbone stiffness of polyrotaxanes can deviate strongly from those of Pst. The higher *M*_w_/*M*_n_ of **3·TM-βCD** and **3·TM-γCD** polyrotaxanes than that of **3** non-rotaxane sample was assigned to the different content of threaded TM-βCD or TM-γCD on the copolymer chains (see incomplete coverage determined by ^1^H NMR).

**Figure 2:**
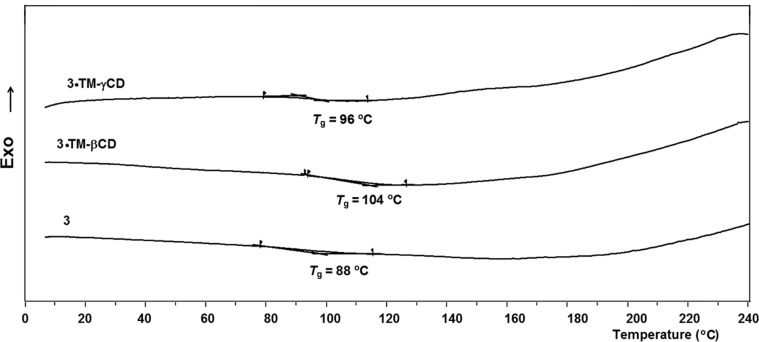
DSC traces on second heating scan of **3**, **3·TM-βCD** and **3·TM-γCD** compounds.

The thermal properties of the copolymers were evaluated by differential scanning calorimetry (DSC) and thermogravimetric analysis (TGA). All copolymers showed only glass-transitions (*T*_g_) and not any exothermal crystallization peak characteristic of polymers containing 9,9-dioctyl-2,7-fluorene units (PFO) [49], Figure 2.

The non-rotaxane copolymer **3** has a *T*_g_ at 88 °C. The *T*_g_ value increases for **3·TM-γCD** and **3·TM-βCD** to 96 °C and 104 °C, with respect to that of the non-rotaxane counterpart. The threading of **1** backbone through the cavities gives a more rigid copolymer structures with increased *T*_g_, as results of its encapsulation, Table 1. It should be mentioned, that increased threading leads to a higher *T*_g_ of the resulting **3·TM-βCD** polyrotaxane. The thermal stability of the copolymers was also investigated by TGA (not shown) and the TGA data revealed that all polymers were stable up to about 300 °C.

**Figure 3:**
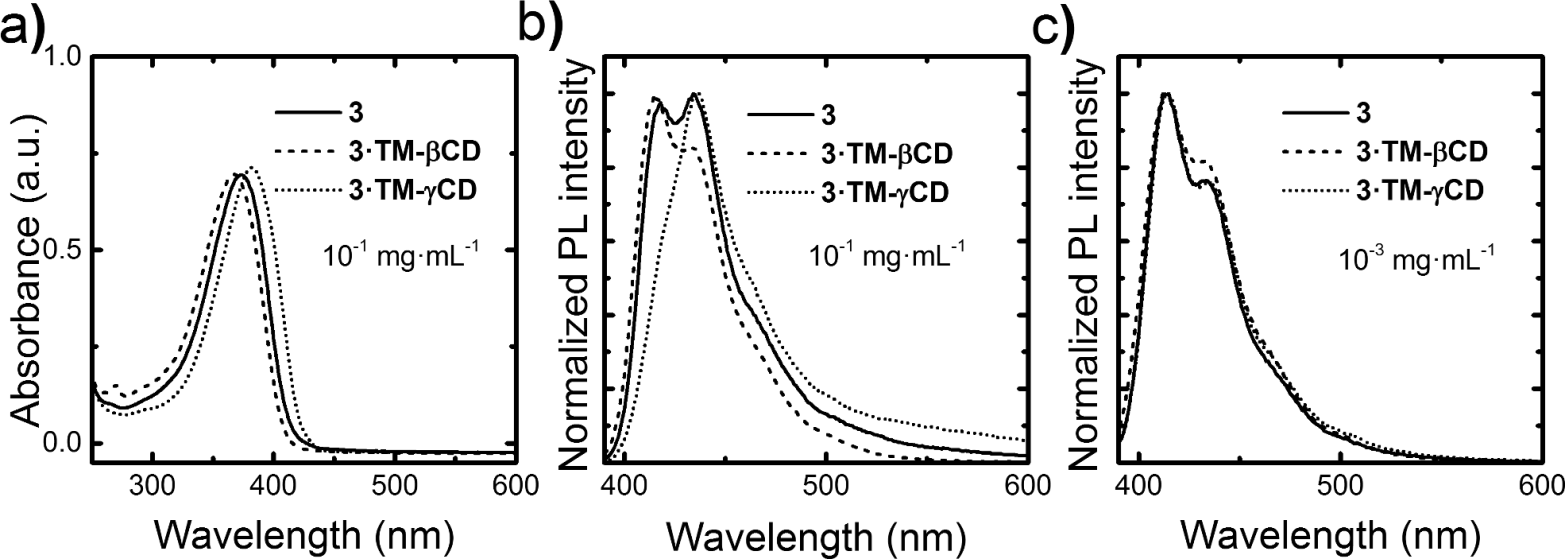
Optical properties of **3·TM-γCD** (dotted line), **3·TM-βCD** (dashed line) and 3 (solid line) polymers: absorption spectra at 10^−1^ mg∙mL^−1^ in CHCl_3_ (a), and normalized emission spectra at 10^−1^ mg∙mL^−1^ and 10^−3^ mg∙mL^−1^ in CHCl_3_, (b) and (c), respectively.

The absorption spectra of **3·TM-βCD** and **3·TM-γCD** polyrotaxanes and the unthreaded **3** counterpart at a concentration of 10^−1^ mg∙mL^−1^ in CHCl_3_ are reported in Figure 3a. The non-rotaxane **3** copolymer shows a featureless band peaking at 374 nm. Upon encapsulation with the TM-βCD, we note a hypsochromic shift of about 7 nm that can be attributed to a reduction of intermolecular interactions and/or a variation of the polarity when the PF core is inside the macrocycles’ cavity. The **3·TM-γCD** polyrotaxane copolymer, instead, displays a red-shift of about 8 nm thereby suggesting the presence of some intrachain species. We consider such a red-shift however, not to be sufficient to infer the presence of fluorenone defects [24], although clear spectroscopic signature of the presence of such species can be gleaned from time-resolved photoluminescence efficiency (PL) experiments.

The PL spectra of the copolymers in CHCl_3_ solutions at a concentration of 10^−1^ mg∙mL^−1^ are reported in Figure 3b. The emission of the non-rotaxane **3** copolymer shows three vibronic components at about 418, 435 and 460 nm. The intensity of the 0–1 fluorescence band (435 nm) for diluted CHCl_3_ solution is the most intense. At the same concentration, **3·TM-βCD** exhibited a slight blue-shift (2 nm) of the emission. The ratio of the emission intensity of the 0–0 transition for **3·TM-βCD** is higher than that of the 0–1 transition, contrary to what we observe for the non-rotaxane **3** counterpart. Such trends suggest that the encapsulation with the macrocycle TM-βCD acts to reduce intermolecular interactions, in agreement with previous reported results [50]. Interestingly, **3·TM-γCD** shows a much stronger 0–1 transition than the 0–0 one, as the non-rotaxane copolymer **3**, which might be indicative of some aggregation even though we do not observe a strong tail in the 500–600 nm regions (apart from the minor red-shift mentioned earlier). We also note that such TM-γCD threaded polyrotaxanes and the unthreaded polymer have a similar PL emissions with the 0–0 the most intense transition for diluted solutions (10^−3^ mg∙mL^−1^ in CHCl_3_), as reported in Figure 3c. It appears that TM-βCDs are much more effective than TM-γCD at suppressing intermolecular interactions upon an increase of the polymer concentration. Such interpretation is also corroborated by the time-resolved PL spectroscopy. Indeed, we find that the temporal decays for the diluted solutions are mono-exponential with a time constant of ≈0.6 ns for the polyrotaxanes and the non-rotaxane polymer at a concentration of 10^−3^ mg∙mL^−1^ in CHCl_3_. However, at a concentration of 10^−1^ mg∙mL^−1^ in CHCl_3_, while the decay is still mono-exponential for **3** and **3·TM-βCD** polymers (τ ≈ 0.7 ns and 0.8 ns, respectively), **3·TM-γCD** polymer shows a bi-exponential decay with τ of 0.7 ns and 5.4 ns, with relative weights of 98 and 2%, respectively. The longer τ for the **3·TM-γCD** polyrotaxane is consistent with “interchain states”. While these do not dominate the luminescence of the materials (the longer lifetime only accounts for 2% of the total PL weight), they are plausible, considered the significantly bigger size of the γCD**,** which might favor both unthreading of the cores, or even accommodation of more than one core unit within the macrocycles cavities. Poor suppression of interchain interactions by γCD had already been observed in the case of diphenylenevinylene rotaxanes, and it is therefore not surprising that we observe similar effects [33].

Interestingly, we measure a photoluminescence quantum efficiency (PLQE) of 66 ± 7% for the **3·TM-γCD**, 56 ± 6% for the **3·TM-βCD** and 46 ± 5% for the reference **3** polymer. Given the relatively large errors in these measurements the only conclusion we can draw is that the unthreaded materials is slightly less efficient than **3·TM-γCD**, but we consider we should not try to read too much into the difference in PL efficiency between **3·TM-γCD** and **3·TM-βCD**.

**Figure 4:**
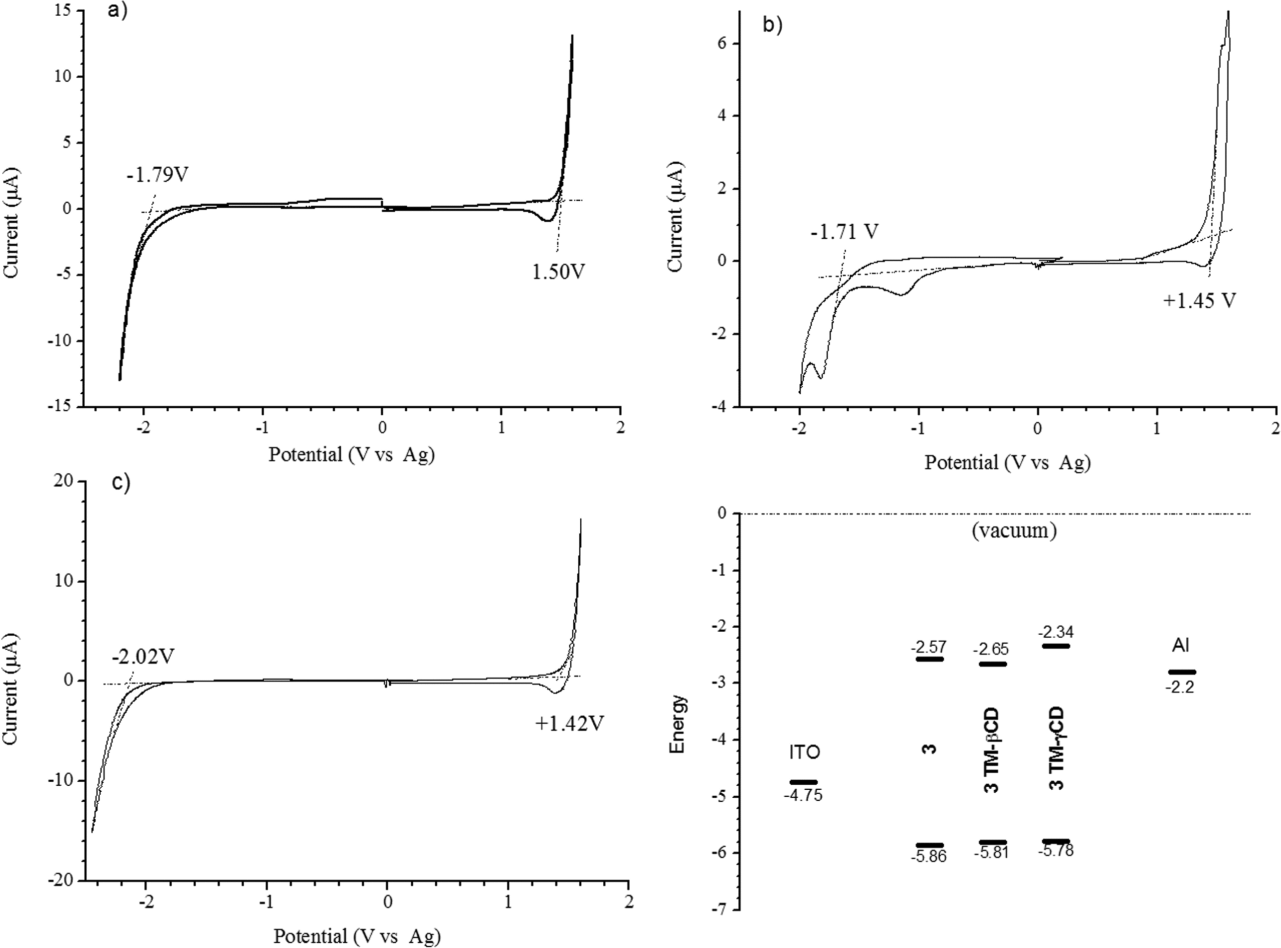
CV of **3** (a), **3·TM-βCD** (b) and **3·TM-γCD** (c) in 0.1 M tetrabutylammonium perchlorate (TBAClO_4_)/ACN solution at scan rate 20 mV∙s^−1^ and HOMO/LUMO energetic levels in addition to the work function of ITO (anode) and Al (cathode) (d).

With a view to understand the factors that control the charge transport within and between conjugated macromolecular chains and the macrocycles, **3**, **3·TM-βCD** and **3·TM-γCD** were electrochemically investigated by cyclic voltammetry (CV), Figure 4 and the results are summarized in Table 2. The *E*_p,onset_ and *E*_n,onset_ values allow the estimation of the ionization potential (IP), electron affinity (EA) and energy band gap (Δ*E*_g_) using ferrocene (Fc) as reference [51]. The IP, EA energy levels and Δ*E*_g_ were calculated according to Equations 1–3 [52–53].

[1]
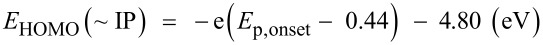


[2]
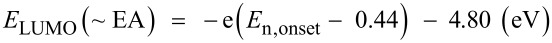


[3]
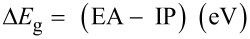


where: −4.80 eV represents the position of the Fc^+^/Fc redox couple in the energetic diagram [51]; +0.44 V is the redox potential of Fc^+^/Fc vs Ag.

**Table 2:** The electrochemical data for **3**, **3·TM-βCD** and **3·TM-γCD** copolymers.

Sample	**3**	**3·TM-βCD**	**3·TM-γCD**

Oxidation^a^ (*E*_p,onset_) (V)	1.5	1.45	1.42
Reduction^b^ (*E*_n,onset_) (V)	−1.79	−1.71	−2.02
*E*_HOMO_ ≈ IP^c^ (eV)	−5.86	−5.81	−5.78
*E*_LUMO_ ≈ EA^d^(eV)	−2.57	−2.65	−2.34
Δ*E*_g_^e^(eV)	3.29	3.16	3.44

^a^Oxidation onset potentials. ^b^Reduction onset potentials. ^c^*E*_HOMO_ = −e(*E*_p,onset_ − 0.44) − 4.80. ^d^*E*_LUMO_ = −e (*E*_n,onset_ − 0.44) − 4.80 (eV). ^e^Electrochemical band gap (Δ*E*_g_ = *E*_LUMO_ − *E*_HOMO_).

As indicated in Table 2, during the *n*-doping process, polyrotaxane **3·TM-βCD** is reduced at a lower potential (−1.71 V) compared to the neat copolymer **3**, and the polyrotaxane **3·TM-γCD**, whose reduction potentials are attained at −1.79 V, and −2.02 V, respectively. The encapsulation of monomer **1** into TM-βCD or TM-γCD cavities appears to have a greater effect on the LUMO energy levels of **3·TM-βCD** and **3·TM-γCD** polyrotaxanes. Furthermore, these results suggest that TM-βCD may impose a more constrictive environment for the monomer **1** than TM-γCD, due to its smaller inner cavity diameter. Consequently there is the possibility for TM-γCD to move along on the monomer **1** backbone, until the stopper groups and these displacements to affect the LUMO energy level of the resulting **3·TM-γCD** polyrotaxane, see Table 2. By contrast, TM-βCD which is more localized on the monomer **1** backbone do not influence the LUMO energy level of **3·TM-βCD** compared to the reference **3**. Obviously, the LUMO energy value is responsible for the low value of Δ*E*_g_ in the case of **3·TM-βCD** polyrotaxane. Note that the redox behaviors of the investigated polyrotaxanes have a similar origin with those of the reference copolymer **3**. Close inspection of the electrochemical results suggest that all three investigated compounds exhibit typical semi-conducting properties, i.e., an insulating behavior in a wide range of potential between *n*- and *p*-doping processes.

As shown by the CV in Figure 4, **3·TM-βCD** exhibited three reduction peaks in the first CV scan at 0.0 V (very small), −1.0 V and at −1.8 V, respectively. The last one corresponds to the *n*-doping process. The peaks from 0.0 V and −1.0 V could be associated with the trapping of ionic charges into the polymer when the polymer returns to its neutral (insulating) state after the first CV scan, as previously reported [20]. Furthermore, these results suggest that the reduction process of **3·TM-βCD** displays a semi-reversible behavior.

The HOMO/LUMO energy levels in combination with the electronic potentials of the anodic indium tin oxide (ITO) glass substrate (−4.75 eV) and cathodic aluminum (−2.2 eV), prove that the investigated compounds are electrochemically accessible as electron-transporting materials for fabrication of organic light-emitting diodes (OLEDs) [54], Figure 4d.

To gain further insights into the effect of macrocyclic encapsulations, it is also important to investigate the influence of the nature of host molecules on the induced chemical changes of the **3·TM-βCD** and **3·TM-γCD** polyrotaxane surfaces. Advancing contact angles (θ) values of water (polar) and diiodomethane (apolar) have been obtained for spin-coated copolymer films, Table 3. The smaller value of θ in water for **3·TM-γCD** (87°) with respect to the non-rotaxane counterpart **3** (100°) reflects its higher hydrophilicity attributed to TM-γCD encapsulation. A different behavior is observed for **3·TM-βCD** which prevented any contact angle measurements. This phenomenon should be attributed to the better dissolution of the spin-coated film of **3·TM-βCD** in water. As can be seen from Table 3, quite similar values were obtained in diiodomethane for the reference **3** and **3·TM-γCD** polyrotaxane. These results are typical of surfaces covered with a close packing of hydrocarbon chains [55]. In contrast, a lower θ value is observed for **3·TM-βCD.** Such phenomenon represents a significant contribution of TM-βCD high coverage.

**Table 3:** Advancing contact angle of water and diiodometane measured on spin-coated film of compounds.

Sample	θ(°)^a^	θ(°)^b^

**3**	100.1 ± 1.9	49.9 ± 0.3
**3**·**TM-βCD**	—^c^	43.5 ± 0.7
**3**·**TM-γCD**	87.3 ± 1.7	48.4 ± 0.8

^a^Water advancing contact angle. ^b^Diiodomethane advancing contact angle. ^c^Due to the dissolution of the spin-coated film, water advancing contact angles prevented any contact angle measurements.

**Figure 5:**
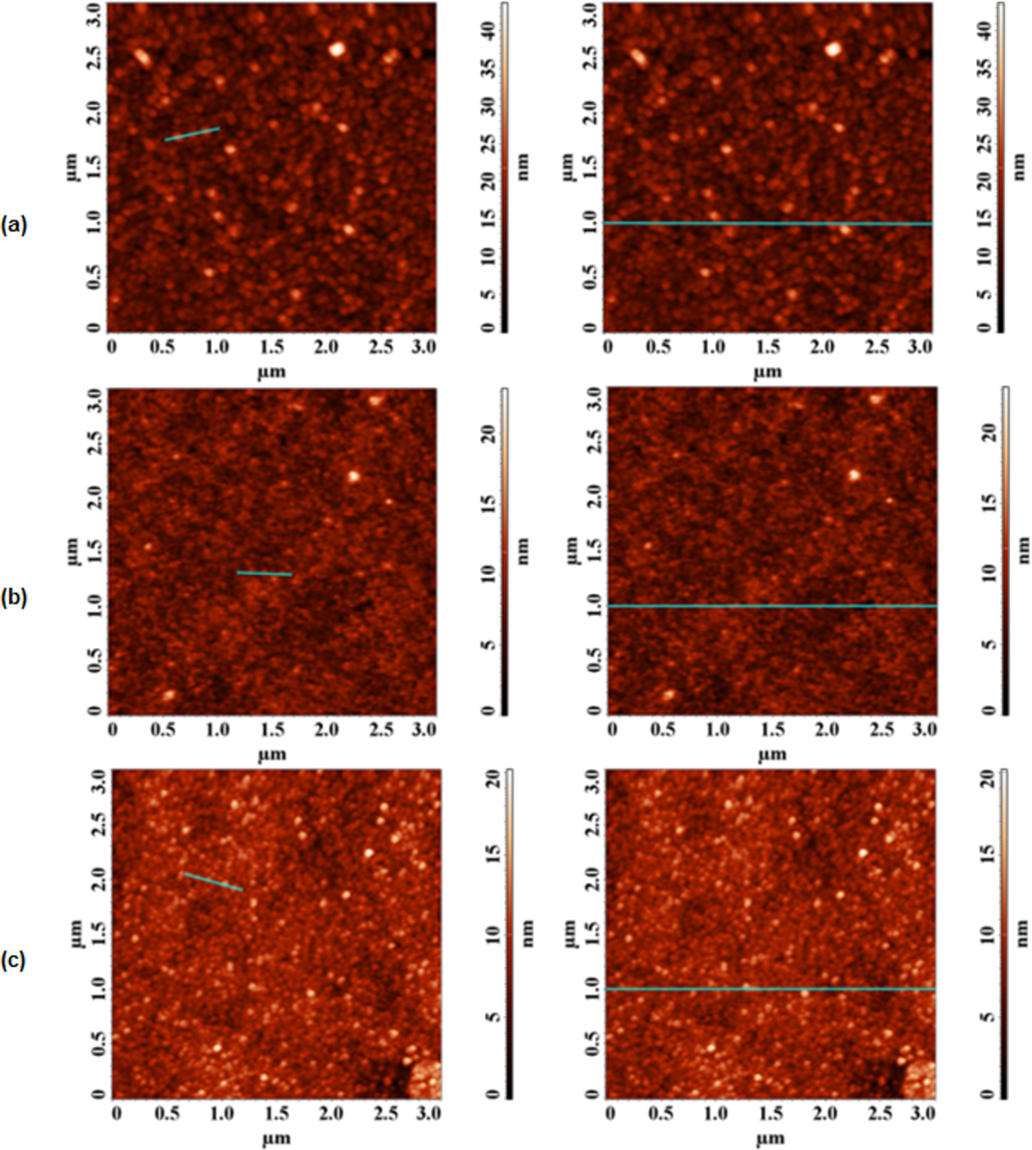
Representative AFM images obtained over 3 × 3 µm^2^ areas of the non-rotaxane **3** (a), **3**·**TM-βCD** (b) and **3**·**TM-γCD** (c) polyrotaxanes.

To further explore the effect of the TM-βCD and TM-γCD encapsulations, the surface topography of the copolymers was also investigated by atomic force microscopy (AFM) analysis. Some representative images obtained for the non-rotaxane **3**, **3·TM-βCD** and **3·TM-γCD** polyrotaxanes over 3 × 3 µm^2^ areas, are shown in Figure 5 and the results are summarized in Table 4.

**Table 4:** Roughness and grains parameters collected from 3 × 3 µm^2^ AFM images of **3**, **3·TM-βCD** and **3·TM-γCD** thin films.

Sample	Surface roughness		
	*Sy* (nm)^a^	*Sq* (nm)^b^	*Sa* (nm) ^c^

**3**	43.7	3.73	2.73
**3·TM-βCD**	21.3	1.76	1.35
**3·TM-γCD**	23.2	1.85	1.42

^a^Peak to valley height. ^b^Root mean square roughness. ^c^Average roughness.

As can be seen from Figure 5, the polyrotaxane film surfaces displayed granular morphologies with lower root mean square roughness *(Sq*) and average roughness (*Sa*) surface parameters compared to that of the non-rotaxane counterpart **3**. It should be note that the lower *Sq* and *Sa* values provide microscopic evidence of the changes in the surface topography of the encapsulated compounds.

Taking into account all the information obtained from AFM analysis, it can be concluded that the lower surface parameters clearly evidenced that the encapsulation with chemically-modified CDs leads to better film forming ability with a smoother surface.

## Conclusion

TM-βCD or TM-γCD encapsulations of PF backbones lead to distinct improvements in the solubility and transparency of the solid films, increased glass-transition temperatures, enhancements of the surface characteristics. The optical investigations confirmed that the encapsulated compounds exhibited higher PLQE and fluorescence lifetimes. These complex architectures showed interesting electrochemical characteristics, which were consistent with optical and surface morphological results. The slightly lower Δ*E*_g_ value for **3·TM-βCD** suggests that the encapsulation have a greater effect on the reduction process, which affects the LUMO values. In addition, HUMO/LUMO energy levels proved that all copolymers are electrochemically accessible in an electroluminescence configuration cell. The present study is significantly valuable and informative as a method to built new conjugated polyrotaxanes by using permodified CD derivatives. Development of new polyrotaxane architectures should be beneficial especially in the field of materials for the generation of active layers in organic electronic devices.

## Experimental

### Materials and methods

**1**, **2**, tetrakis(triphenylphosphine)palladium(0) [Pd(PPh_3_)_4_], β- and γCD, bromobenzene (Br–Ph), dimethylformamide (DMF), dimethyl sulfoxide (DMSO), and quinine sulfate dehydrate in 0.5 M sulfuric acid were purchased from (Sigma-Aldrich) and used as received. TBAClO_4_ for electrochemical analysis (99.0%) (Fluka) was used without further purification. Acetonitrile (ACN) (Fischer), DCM, CHCl_3_, toluene and all other solvents were purchased from commercial sources (Sigma-Aldrich, Fisher) and used without further purification.

^1^H NMR spectra have been recorded on a Bruker Avance DRX 400 MHz instrument equipped with a 5 mm QNP direct detection probe and z-gradients. Spectra have been recorded in CDCl_3_ at room temperature. The chemical shifts are reported as δ values (ppm) relative to the residual peak of the solvent. The FTIR (KBr pellets) spectra were obtained on a Bruker Vertex 70 spectrophotometer. The molecular weights of copolymers were determined by GPC in THF by using a Water Associates 440 instrument and polystyrene (Pst) calibrating standards. DSC was performed with a Mettler Toledo DSC-12E calorimeter with two repeated heating–cooling cycles at a heating rate of 5 °C·min^−1^ under N_2_ atmosphere. TGA analysis was performed under constant nitrogen flow (20 mL·min^−1^) with a heating rate of 10 °C·min^−1^ using a Mettler Toledo TGA/SDTA 851e balance. UV–vis and fluorescence spectra in CHCl_3_ solutions were performed using **3**, **3·TM-βCD** and **3·TM-γCD** with the same concentration (either 10^−1^ mg∙mL^−1^ or 10^−3^ mg∙mL^−1^) of the **3** cores without macrocyclic molecules. Time-resolved photoluminescence (PL) measurements were performed with a time-correlated single photon counting (TCSPC) spectrometer previously reported [17]. The PLQE was estimated by comparison with a solution of quinine sulfate dehydrate in 0.5 M sulfuric acid of known quantum efficiency, 56 ± 5%.

CVs were carried out in a three-electrode cell in which Pt (1 mm diameter) was used as a working electrode, a Pt wire as counter-electrode and an Ag wire as pseudo-reference electrode. A TBAClO_4_ solution (0.1 M) in anhydrous ACN was used as the supporting electrolyte. The set-up was introduced into a glove box and controlled by AUTOLAB PGSTAT 101 (Ecochemie) using NOVA software. The pseudo-reference was calibrated with a 10^−3^ M of Fc solution in ACN. The polymer samples were drop-casted onto the working electrode from a concentrated DCM solution and studied in the interval −2.5 and +2.0 V vs Ag wire. Cathodic and anodic scans were performed independently.

The surface profiles of copolymers films were evaluated by AFM measurements. AFM were performed in the tapping mode, using a Solver PRO-M scanning probe microscope (NTMDT, Russia) with commercially available NSG10 cantilever. Films were prepared onto mica substrates by spin-coating from CHCl_3_ solution at 3000 rpm for 60 s on a WS-400B-6NPP-Lite Single Wafer Spin Processor (Laurel Technologies Corporation, USA). Scan areas of 3 × 3 μm^2^, were analyzed with a resolution of 512 × 512 pixels. Advancing and receding contact angle measurements were performed by using the drop shape analysis profile device equipped with a tiltable plane (DSA-P, Kruss, Germany). Ultrapure water (Millipore, resistivity = 18 MΩ·cm) or a diiodomethane drop was first deposited on the sample using a variable volume micropipette. The drop volume was set to 15 µL for water and 10 µL for diiodomethane. In order to perform dynamic contact angle measurements, the sample surface sustaining the drop was tilted at a constant speed (1 deg·s^−1^) and the images of the drop simultaneously recorded. The advancing contact angle was measured at the front edge of the drop, just before the triple line starts moving. The angle was obtained using the tangent of the drop profile at the triple line. For each sample, contact angles were measured on four samples and three drops per sample. The reported contact angle values correspond to the average of all measurements with an error bar corresponding to the standard deviation.

**Synthesis of 2,3,6-tri-*O*-methyl-CD (TM-βCD) and 2,3,6-tri-*O*-methyl-CD (TM-γCD):** TM-βCD and TM-γCD as macrocyclic molecules were synthesized according to previously reported procedure [47–48].

**Synthesis of 1**·**TM-βCD:** To prepare **1·TM-βCD** inclusion complex, 0.572 g (0.4 mmol) of TM-βCD were dissolved in water (5.0 mL) and 0.067 g (0.2 mmol) of **2** were added. The mixture was stirred at room temperature under nitrogen atmosphere for 48 h to give a turbid dispersion. The water was removed by lyophilization and the complex, as a white powder was used for the preparation of **1·TM-βCD**. The synthesis of the inclusion complex **1·TM-γCD** was performed under similar experimental conditions as those used for the preparation of the **1·TM-βCD** inclusion complex, except (0.654 g, 0.4 mmol) of TM-γCD was used instead of TM-βCD.

**Synthesis of 3**·**TM-βCD and 3**·**TM-γCD polyrotaxane copolymers: 1·TM-βCD** (0.639 g, 0.2 mmol) and **2** (0.115 g, 0.2 mmol) were dissolved into 6 mL of toluene in a flask under argon (Ar) protection. The mixture was flushed with Ar several times, and then 1.5 mL of a 3 M solution of sodium carbonate (Na_2_CO_3_) and 18.2 mg of (Ph_3_P)_4_Pd(0), as catalyst dissolved in 4 mL of degassed toluene were added into the flask. The solution was flushed with Ar again for another three times, and the reaction mixture was protected against light. The oil bath was heated to 90 °C, and the reaction mixture was stirred for 72 h. Then, an excess of 0.005 g (0.01 mmol) of monomer **2** dissolved in 3 mL of toluene was added and the reaction was continued for 12 h. Finally, 1.0 μL of Br-Ph was added as end-capper of the copolymer chain and the reaction was continued overnight. After cooling, the mixture was poured into water and extracted with toluene. The organic extracts were washed with water and dried over magnesium sulfate (MgSO_4_). The toluene solution was concentrated by rotary evaporation and precipitated in CH_3_OH. The solid was filtered, dried and purified by Soxhlet extraction with methanol and acetone in succession to remove the oligomers. The polymer was further purified by reprecipitation from concentrated CHCl_3_ solution with methanol, collected by centrifugation and vacuum dried at 60 °C to afford **3·TM-βCD** (128 mg, 18.8% yield) as a yellow-brownish solid. ^1^H NMR (400 MHz, CDCl_3_) 7.92–7.55 (m, Ha–d and a’–d’), 6.85–6.81 (m, Ph), 5.13 (d, *J* = 3.6 Hz, 7H, C(1)H), 4.11–4.09 (m, Hg’), 3.88–3.80 (m, 14H, C(5)H, C(6)H), 3.65 (s, 21H, O(3’)-CH_3_), 3.62–3.58 (m, 14H, C(4)H, C(6)H), 3.54–3.49 (m, 28H, C(3)H, O(2’)-CH_3_), 3.39 (s, 21H, O(6’)-CH_3_), 3.19 (dd, *J* = 3.6 Hz, 7H, C(2)H), 2.10–1.93 (m, Hh), 1.26–1.11 (m, Hi-n), 0.83–0.71 (m, Ho); FTIR (KBr, cm^–1^): 3433, 2927, 2853, 1724, 1614, 1459, 1410, 1357, 1159, 1091, 1042, 968, 875, 813 cm^−1^; GPC (THF, Pst standard): *M*_n_ = 24300 g·mol^−1^, *M*_w_/*M*_n_ = 1.94.

**3·TM-γCD** was synthesized by similar experimental conditions as described for **3·TM-βCD**, except that TM-γCD was used instead of TM-βCD. **3·TM-γCD** polyrotaxane was also obtained as a yellow-brownish solid in a 24.7% yield. ^1^H NMR (400 MHz, CDCl_3_) 7.92–7.38 (m, Ha–d and a’–d’), 6.91 (s, Ph), 5.26–5.02 (m, 7H, C(1)H), 4.1–3.25 (m, Hg’, C(2–6)H, O(2’,3’,6’)-CH_3_), 2.11 (s, Hh), 1.11 (s, Hi–n), 0.81 (s, Ho); ^13^C NMR (100 MHz, CDCl_3_) 151.79–140.10 (C c, e, f, c’, e’, f’), 132.18–120.21 (C a, b, d, a’, b’, d’), 98.09 (C1), 82.11 (C2, 4), 71.04 (C5,6 ), 61.07–59.02 (C 2’, 3’, 6’), 55.41 (C g), 40.49 (C h), 37.18 (Cg’), 31.79 (Ci), 30.05–29.20 (Cj–m), 22.59 (Cn), 14.02 (Co); FTIR (KBr, cm^–1^): 3416, 3058, 2923, 2850, 1634, 1610, 1457, 1405, 1373, 1291, 1095, 888, 810, cm^−1^; GPC (THF, Pst standard): *M*_n_ = 20100 g·mol^−1^, *M*_w_/*M*_n_ = 2.24.

**Synthesis of the non-rotaxane 3 copolymer:** The non-rotaxane copolymer **3** was synthesized under similar experimental conditions as those described for **3·TM-βCD** or **3·TM-γCD** polyrotaxanes, except that free monomer **1** was used instead of **1·TM-βCD** or **1·TM-γCD**. The crude polymer **3** was collected by filtration and then extracted with a Soxhlet extractor using methanol and acetone. Further the solid was redissolved in CHCl_3,_ precipitated with methanol, collected by filtration and vacuum dried at 50 °C. The copolymer was obtained as an orange solid in a yield of 47.8%. ^1^H NMR (400 MHz, CDCl_3_) 7.98–7.39 (m, Ha–d and a’–d’), 6.93–6.87 (m, Ph), 4.14–4.06 (m, Hg’), 2.16 (s, Hh), 1.16 (s, Hi–n), 0.86 (s, Ho); ^13^C NMR (100 MHz, CDCl_3_) 151.77–140.05 (C c, e, f, c’, e’, f’), 128.79–120.24 (C a, b, d, a’, b’, d’), 55.37 (Cg), 40.47 (Ch), 37.18 (Cg’), 31.79 (Ci), 30.04–29.21 (Cj–m), 22.60 (Cn), 14.06 (Co); FTIR (KBr, cm^–1^): 3438, 3024, 2954, 2922, 2850, 1605, 1457, 1405, 1378, 1261,1196, 1092, 1023, 810 cm^−1^; GPC (THF, Pst standard): *M*_n_ = 27900 g·mol^−1^, *M*_w_/*M*_n_ = 1.83.

## Supporting Information

File 1 Characterization data of the compounds: The stability constant, FTIR, ^1^H NMR and ^13^C NMR spectra of the investigated copolymers.
