# Supplementary material for: Synthesis and photophysical characteristics of polyfluorene polyrotaxanes
Source: Beilstein J Org Chem. 2015 Dec 21;11:2677–88. doi: 10.3762/bjoc.11.288 (PMC4734422; doi:10.3762/bjoc.11.288)
Supplement: File 1 — Characterization data of the compounds: The stability constant, FTIR, 1H NMR and 13C NMR spectra of the investigated copolymers. [file Beilstein_J_Org_Chem-11-2677-s001.pdf]

Supporting Information

for

Synthesis and photophysical characteristics of polyfluorene polyrotaxanes

Aurica Farcas*[,1], Giulia Tregnago[2], Ana-Maria Resmerita[1], Pierre-Henri Aubert[3] and Franco Cacialli[*,2]

Address: [1] Supramolecular Chemistry Group, ‘‘Petru Poni’’ Institute of Macromolecular Chemistry, Grigore Ghica Voda Alley, 700487-Iasi, Romania, [2]London Centre for Nanotechnology and Department of Physics and Astronomy University College London, Gower Street, London WC1E 6BT, UK and [3]Laboratoire de Physicochimie des Polymères et des Interfaces (EA 2528), Institut des Matériaux, Université de Cergy-Pontoise, F-95031 Cergy-Pontoise Cedex, France

Email: Franco Cacialli - f.cacialli@ucl.ac.uk; Aurica Farcas - afarcas@icmpp.ro

*Corresponding authors

Characterization data of the compounds: The stability constant, FTIR, ^{1}H NMR and ^{13}C NMR spectra of the investigated copolymers

Table of contents for Supporting Information **page**

The stability constant of the guest **1** towards the host TM-βCD s2

The stability constant of the guest **1** towards the host TM-γCD s2

FTIR spectra of the reference **3**, **3·TM-βCD** and **3·TM-βCD** polyrotaxanes s4

^{1}H NMR spectrum of the non-rotaxane **3** copolymer s5

^{13}C NMR of the non-rotaxane **3** copolymer s6

^{1}H NMR spectrum of the polyrotaxane **3·TM-γCD** copolymer s7

^{13}C NMR of the polyrotaxane **3·TM-γCD** copolymer s8

References s8

S1. The stability constant (K_s) of **1·TM-βCD** inclusion complex

Figure S1 shows the changes in the absolute optical density (OD) at 321 nm with increasing concentration of TM-βCD to a 2 μM solution of **1** in $CHCl_3$. The data were obtained by using a direct binding equation, according to a 1:1 host–guest complexation stoichiometry in ProFit 6.2 (QuantumSoft) [1]. The determined value was found to be 580 (±100) M^{-1} for **1·TM-βCD** inclusion complex. The value is in good accordance with the reported stability constants of CDs with different guest molecules in organic solvents [2]. To our knowledge, K_s of monomer **1** encapsulated inside of TM-βCD or TM-γCD has not been studied yet.

S2. The stability constant (K_s) of **1·TM-γCD** inclusion complex

K_s was determined by measuring the change in the absolute optical density (OD) at 321 nm with increasing concentration of TM-γCD and the fitting, according to a 1:1

host-guest complexation stoichiometry, Figure S2. The determined value was found to be 160 ± 30 M^{-1} lower than that of **1·TM-βCD** inclusion complex, due to its larger cavity which allows better dethreading of the guest **1**. This part of our studies provided us the unexpected better results concerning the binding affinity of monomer **1** compared to 5,5'-dibromo-2,2'-bithiophene, as previously reported [3,4].

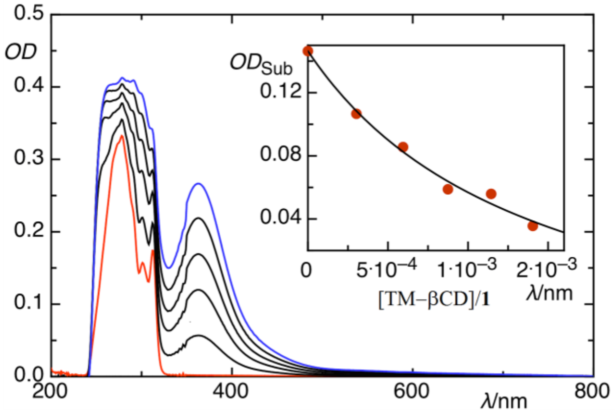

Figure S1: The change in the absorption spectra upon addition of TM-βCD to a 2 μM solution of **1** in $CHCl_3$ at λ_{ex} = 321 nm. The fitted binding constant curve (according to a 1:1 host-guest complexation stoichiometry) is shown in the inset.

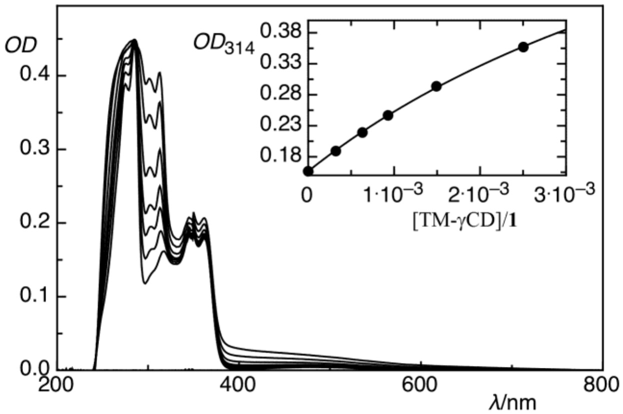

Figure S2: The change in the absorption spectra upon addition of TM-γCD to a 3 μM solution of **1** in $CHCl_3$ at λ_{ex} = 321 nm. The fitted binding constant curve (according to a 1:1 host–guest complexation stoichiometry) is shown in the inset.

S3. FTIR spectra of the reference **3**, **3·TM-βCD** and **3·TM-γCD** polyrotaxane copolymers

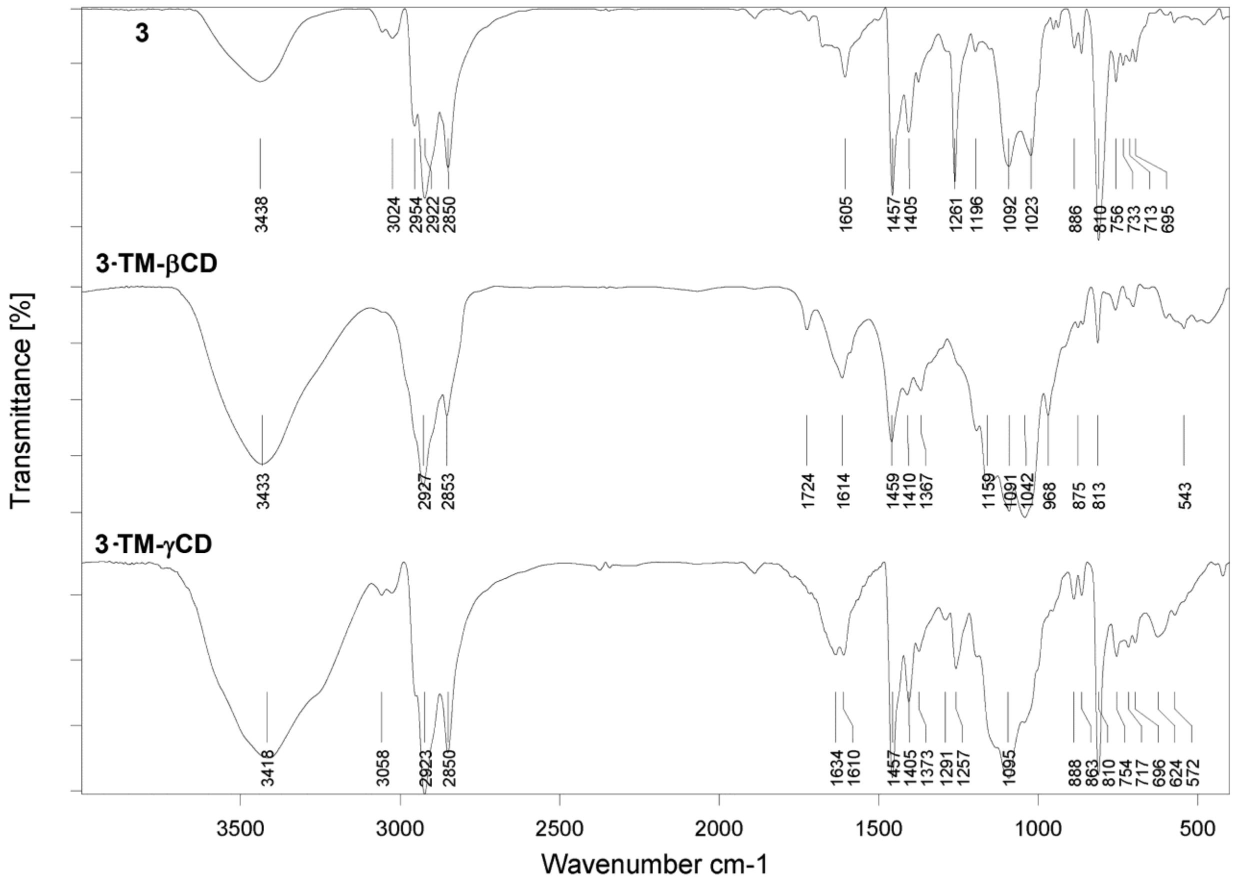

Figure S3: FTIR spectra of the reference **3**, **3·TM-βCD** and **3·TM-γCD** polyrotaxane copolymers

Figure S3 compares the FTIR spectra of **3**, **3·TM-βCD** and **3·TM-γCD** polyrotaxane copolymers. FTIR spectrum of the non-rotaxane **3** exhibited characteristics stretching vibrations in the 2954–2850 cm^{-1} region (aromatic β C–H and ϕ C–H stretching) that are shifted towards lower frequency in the spectra of **3·TM-βCD** and **3·TM-γCD** polyrotaxanes. FTIR spectra of **3·TM-βCD** and **3·TM-γCD** show all the characteristic bands of **3** and additional bands located in the 1159–1042 cm^{-1} region corresponding to TM-βCD and TM-γCD.

S4. ^{1}H NMR spectrum of the non-rotaxane **3** copolymer

The ^{1}H NMR and ^{13}C NMR spectra, with resonance peak assignments of the non-rotaxane **3** confirmed its chemical structure, Figures S4 and S5.

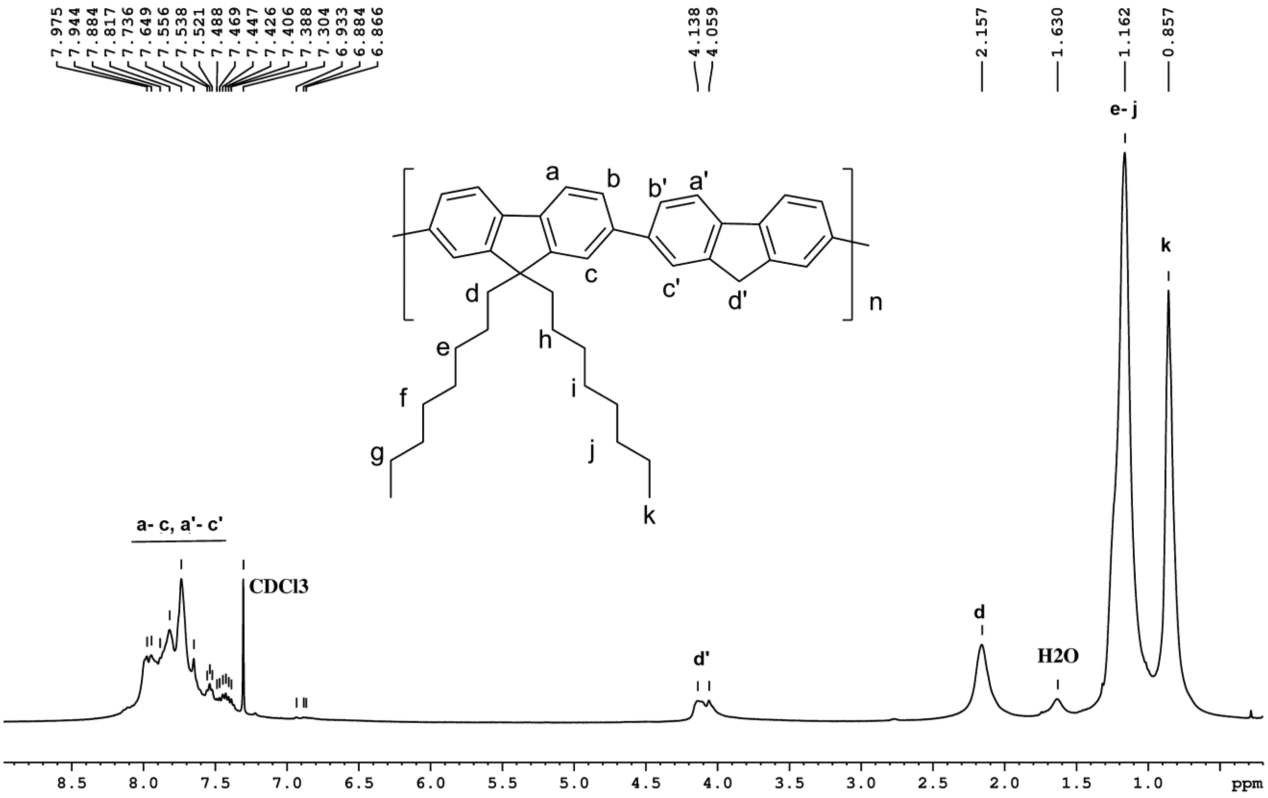

Figure S4: ^{1}H NMR spectrum of the non-rotaxane **3** copolymer with assignments of the resonance peaks in $CDCl_3$.

S5. ^{13}C NMR of the non-rotaxane **3** copolymer

As shown in Figure S4, all typical proton resonance peaks of the non-rotaxane copolymer are visible in the spectrum in $CDCl_3$.

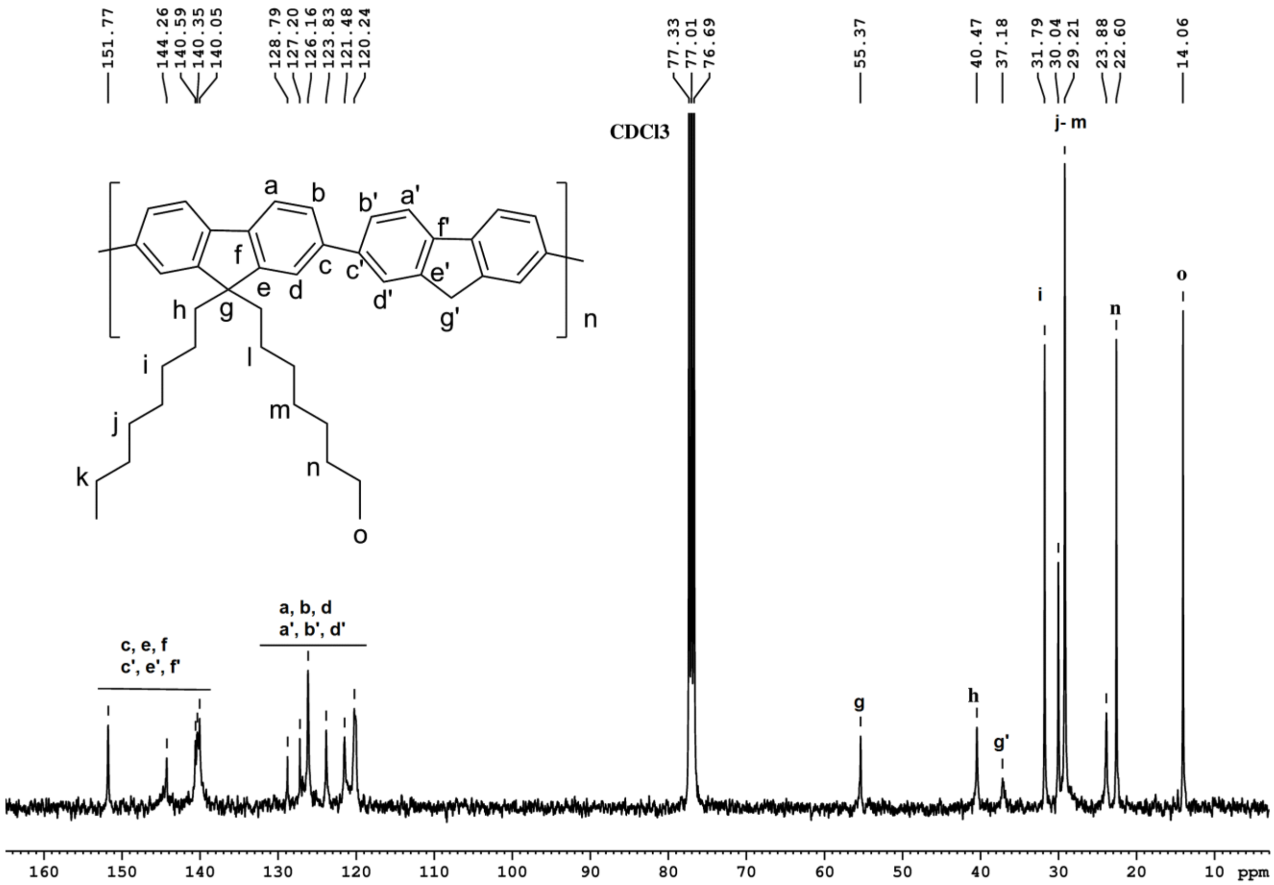

Figure S5: ^{13}C NMR spectrum of the non-rotaxane **3** copolymer with assignments of the resonance peaks in $CDCl_3$.

S6. ^{1}H NMR spectrum of the polyrotaxane **3·TM-γCD** copolymer

The ^{1}H NMR analysis was used to determine the coverage of the rotaxane with macrocycle, i.e., the average number of TM-γCD macrocycles per repeating unit, which has been calculated using the ratio of the integrated area of the peak assigned to the aromatic protons labeled “d” in Figure S6 (2.106 ppm, I_d) and the anomeric H-1 proton of TM-γCD (5.18–5.26 ppm, I_{H^1}); (I H^d/4)/(I H^1/8). The average number of TM-γCD macrocycles per monomer **1** repeat unit has been found to be 0.11 (i.e., 11% coverage). It should be noted that the determined coverage is in accordance with the reported value

of the stability constant, and suggested that much more dethreading or equilibration had occurred compared with TM-βCD under the conditions of the polycondensation reaction.

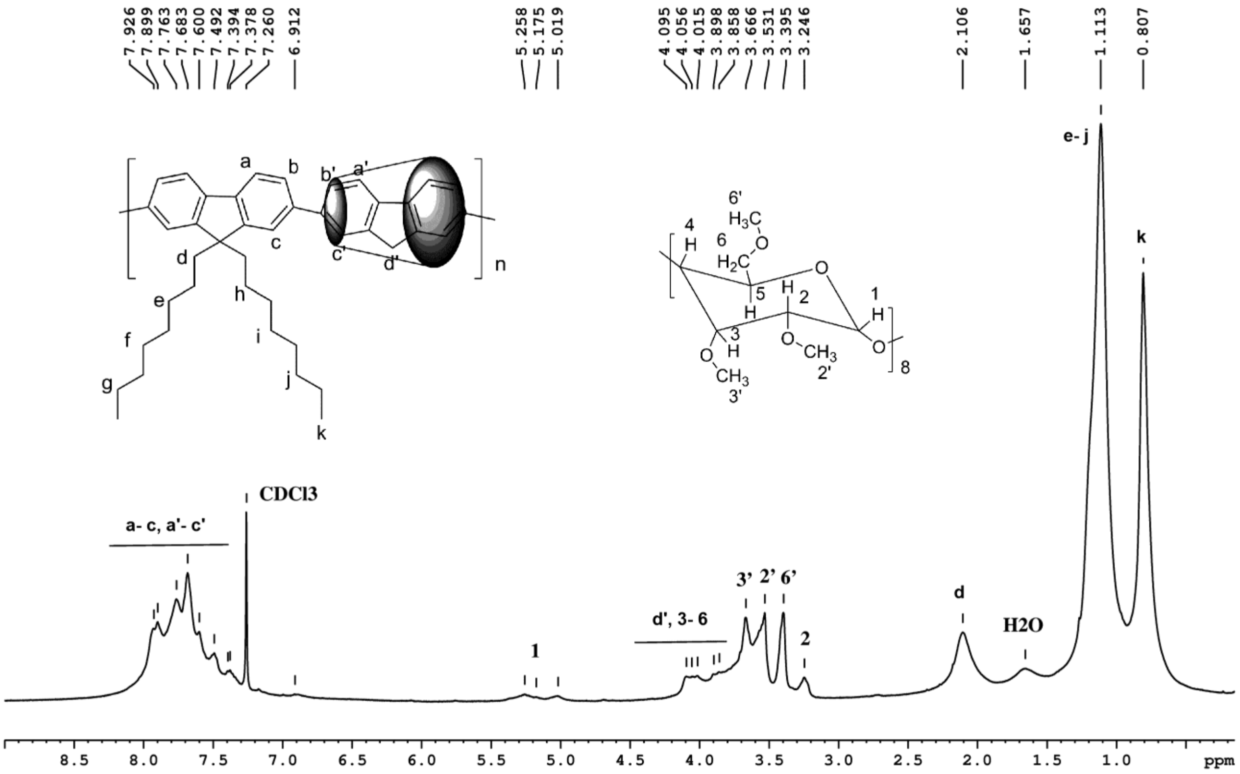

Figure S6: ^{1}H NMR spectrum of the polyrotaxane **3·TM-γCD** copolymer in $CDCl_3$ with assignments of the resonance peaks.

S7. ^{13}C NMR of the non-rotaxane **3·TM-γCD** polyrotaxane

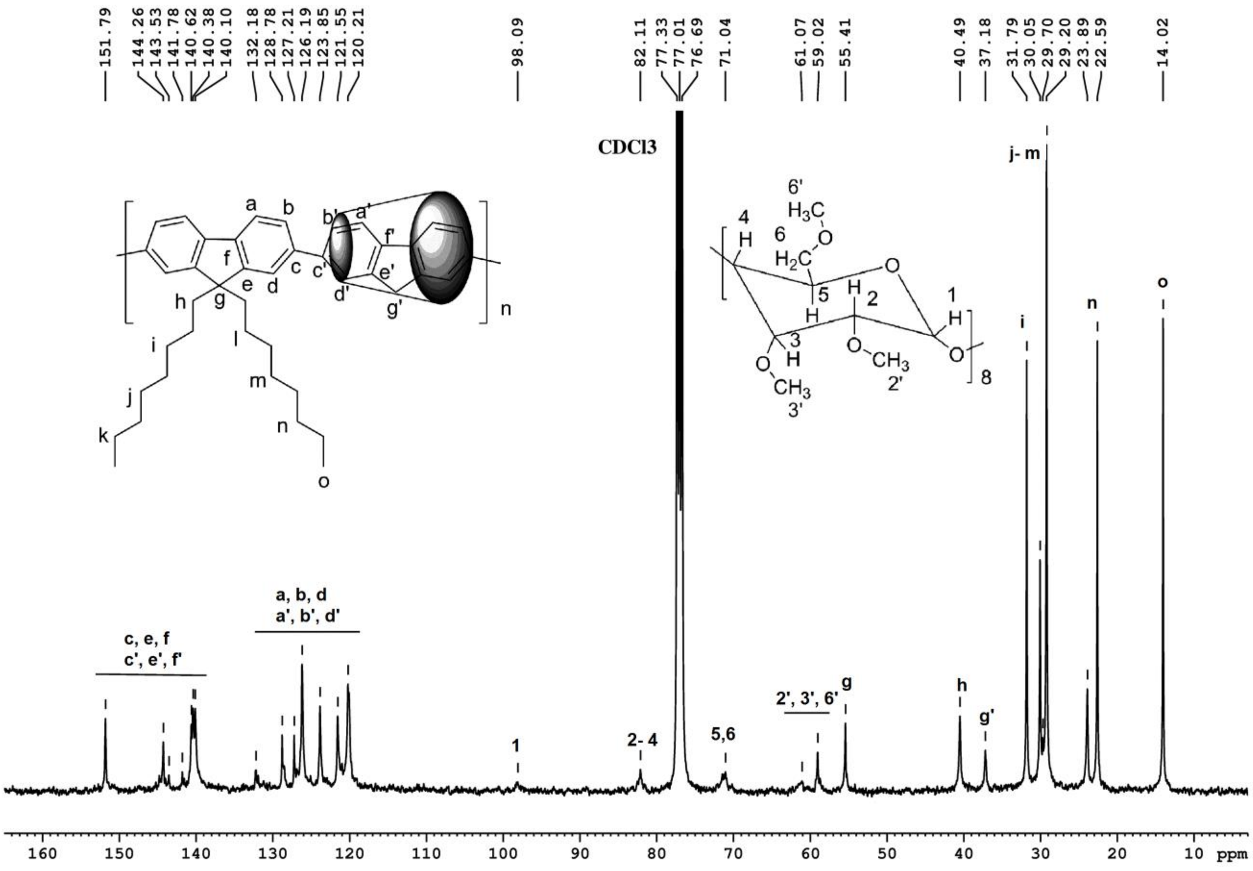

Figure S7: ^{13}C NMR spectrum of the non-rotaxane **3·TM-γCD** copolymer with assignments of the resonance peaks in $CDCl_3$.

References

[1] Nau, W. M.; Zhang, X. Y.; *J. Am. Chem. Soc.* **1999**, *121*, 8022-8032.

[2] Rekharsky, M. V.; Inoue, Y. *Chem. Rev.* **1998**, *98*, 1875-1918.

[3]. Farcas, A.; Ghosh, I.; Grigoras, V. C.; Stoica, I.; Peptu, C.; Nau, W. M. *Macromol. Chem. Phys.* **2011**, *212*, 1022–1031.

[4]. Stefanache, A.; Silion, M.; Stoica, I.; Fifere, A.; Harabagiu, V.; Farcas, A. *Eur. Polym. J.* **2014**, *50*, 223-234
